# Construct validity and internal consistency of Hall’s Professionalism Scale: tested on South African nurses

**DOI:** 10.1186/s13104-019-4515-6

**Published:** 2019-08-06

**Authors:** Tinda Rabie

**Affiliations:** NuMIQ Focus Area, School of Nursing Sciences, Faculty of Health Sciences, North-West University (Potchefstroom Campus), Private Bag X6001, Potchefstroom, 2520 South Africa

**Keywords:** Attitudes, Professionalism, Professional nurses, Nursing practice environments

## Abstract

**Objective:**

In South Africa, appropriate criteria to measure the professional standing of professional nurses are essential. Internationally, there are professionalism scales by which to measure professionalism, but none could be identified that were particular to the South African context. Hall’s Professionalism Scale consists of 50 items and was specifically developed to measure the attitudes and ideologies held by professionals in various professional occupations by measuring five attitudinal components of professionalism, namely: Sense of calling to the field; Autonomy; Using professional organisation as major referent; Belief in self-regulation; and Belief in public service. In this study, the construct validity and internal consistency of the constructs of Hall’s Professionalism Scale were assessed among professional nurses in the South African context.

**Results:**

Originally Hall’s Professionalism Scale comprises 50 items. This scale was reassessed by Snizek, who retained only 25 items of the original scale to measure professional standing. During preliminary analysis of the South African data, 23 items were included.

**Electronic supplementary material:**

The online version of this article (10.1186/s13104-019-4515-6) contains supplementary material, which is available to authorized users.

## Introduction

In South Africa, there are few studies that focus on professionalism amongst nurses, and there is no well-known instrument by which to gauge professionalism. However, many international studies have used Hall’s Professionalism Scale (HPS) to measure professionalism [1]. This scale was developed by Professor Richard Hall, who aimed to determine suitable criteria and measure a person’s professional standing [2]. Professional standing refers to a person belonging to a regulated profession—such as South African professional nurses who are regulated by the South African Nursing Council—to validate the veracity and legitimacy of a person in a particular profession [3]. The 50-point Likert scale measures professionalism in 5 domains [2], namely: the use of a professional organisation as major referent, belief in public service, belief in self-regulation, sense of calling to the field, and autonomy. The Likert scale options are: very well (VW), well (W), neutral opinion (?), poorly (P) and very poorly (VP) [4]. The first domain ‘Using a professional organisation as major referent’, refers to the importance of being affiliated with the professional community to ensure good standards, values and principles in the profession [1, 4]. This includes having a sense of professional commitment to attend meetings, keeping abreast of developments in the field, and being willing to support and participate in committees [5]. Secondly, the ‘Belief in public service’ focuses on whether professionals believe that their profession is beneficial and indispensable to the communities they serve [4]. In some professions such as nursing, the public sometimes does believe in the indispensability of certain services delivered, resulting in professionals in a particular profession to be slow to develop this belief themselves [5]. Thirdly, ‘Belief in self-regulation’ refers to the “professional endorsement of the notion of colleagues’ control”, meaning that a person only feels comfortable if their work is judged by other professionals in the same field, and not by outsiders [4, 5]. The fourth domain, ‘Sense of calling to the field’, focuses on the dedication and commitment of a professional to his/her work. The professional person is motivated by a higher purpose than mere financial gain; there is an ‘inward calling’ [4, 5]. The last item is ‘Autonomy’, refers to the practitioner’s ability to make their own decisions and render judgements about services independent of outside pressures [4, 5]. Hall originally tested 50-item scale on 328 participants, including nurses Cronbach’s alpha was 0.86 and Snizek’s shorter version which included 25 of the original 50 items yielded a Cronbach’s alpha of 0.79 [4].

## Main text

### Methods

A preliminary study with a quantitative, cross-sectional survey design was conducted. All-inclusive sampling (N = 2 158; n = 166) of professional nurses who were enrolled or completed their under and/or post graduate studies (2010–2017) at a particular university were included. Data collection took place via Survey Monkey, an online survey platform. Participants were invited to participate in the study via a computerized short message system (SMS) that communicated the following: “Dear Mr./Mrs./Miss, you are invited to participate in a web-based 50 item survey based on the professionalism of nurses in nursing practice environments in South Africa. Should you decide to participate, kindly follow the http://-link”. This message was sent out on three different occasions (days 1, 2 and 6) in 2017. If respondents opened the http://-link, they were directed to an introductory page and informed consent form, if they ticked the agreement box on the screen, they continued to the questionnaires consisting of two sections (Section A—demographic profile and Section B—the 50 items of HPS). Data was analysed by means of the SPSS Version 25 computer program and included descriptive statistics for the demographic profile and exploratory and confirmatory factor analyses to determine construct validity of HPS.

### Results

#### Demographic profile

Of the 166 (N) professional nurses, 19 (11.45%) were male and 147 (88.55%) female. Forty-one (24.70%) were younger than 30 years, 49 (29.52%) were younger than 39, 52 (31.33%) were younger than 49 years, and 24 (14.45%) were 50 years and older. 109 of the nurses (65.66%) had diplomas in nursing and 57 (34.34%) had baccalaureate degrees. Ninety-eight (59.04%) were employed in the public and 68 (40.96%) in the private health sector. Of the nurses, 158 (95.18%) were employed on a full-time and 8 (4.82%) part-time.

#### Construct validity and reliability

##### Exploratory factor analysis

The results of the Varimax and Oblimin rotations were very similar, confirming literature reports that the type of rotation used should not have a significant influence. Finally, Oblimin rotations were accepted because there were meaningful correlations between some factors. Loadings of below 0.30 were suppressed; however, smaller loadings of items of the original HPS were included in order to include as many items as possible (Table 1).

**Table 1:** Exploratory factor analysis

Factors	Items		Factor 1: Sense of calling to the field	Factor 2: Autonomy	Factor 3: Using the professional organisation as major referent	Factor 4: Belief in self-regulation	Factor 5: Belief in public service
Factor 1: Sense of calling to the field	4	A person enters this profession because he likes the work	*0.520*				
	9	People in this profession have a real “calling” for their work	*0.541*				
	14	The dedication of people in this field is most gratifying	*0.496*	0.326			
	19	Professional training itself helps assure that people maintain their high ideals	*0.304*	0.366			0.306
	24	It is encouraging to see the high level of idealism which is maintained by people in this field	*0.240*	0.523			
	29	Although many people talk about their high ideals, very few are really motivated by them					0.535
	34	It is hard to get people to be enthusiastic about their work in this field			− 0.482	− 0.349	
	39	Most people would stay in the profession even if their incomes were reduced	*0.293*				− 0.531
	44	Most of the real rewards of my work can’t be seen by an outsider					0.332
	49	There are very few people who don’t really believe in their work		0.453			
Factor 2: Autonomy	5	I make my own decisions in regard to what is to be done in my work	0.556				
	10	It is easier when someone else takes responsibility for decision making		*0.644*			
	15	I don’t have much opportunity to exercise my own judgment	− 0.398	*0.438*		− 0.318	
	20	I know that my own judgment on a matter is the final judgment	0.418			− 0.322	
	25	The fact that someone checks your decisions makes this work easier		*0.576*			
	30	When problems arise at work, there is little opportunity to use your own intellect		*0.325*	− 0.448	− 0.364	
	35	There is little autonomy in this work				− 0.518	
	40	My own decisions are subject to review		*0.288*	− 0.451		
	45	I am my own boss in almost every work-related situation	0.495				
	50	Most of my decisions are reviewed by other people		*0.513*			
Factor 3: Using the professional organisation as a major referent	1	I systematically read the professional journals			*0.553*		
	6	I regularly attend professional meetings at the local level	0.485				
	11	I enjoy seeing my colleagues because of the ideas that are exchanged	0.495				0.303
	16	I believe that the professional organization(s) should be supported	–	–	–	–	–
	21	The most stimulating periods are those spent with colleagues	0.462	0.313			
	26	The professional organization doesn’t really do too much for the average member	0.301				− 0.470
	31	The real test of how good a person is in his field is the layman’s opinion of him				− 0.398	
	36	Although I would like to, I really don’t read the journals too often			*0.547*		
	41	Most of my own friends are not fellow professionals	–	–	–	–	–
	46	The profession doesn’t really encourage continued training			*0.512*		
Factor 4: Belief in self-regulation	3	A person who violates professional standards should be judged by his professional peers	0.369			− *0.229*	
	8	My fellow professionals have a pretty good idea about each other’s competence	0.395				
	13	There really aren’t any penalties for the person who violates professional standards				− *0.387*	
	18	A problem in this profession is that no one really knows what his colleagues are doing				− *0.547*	
	23	A basic problem for the profession is the intrusion of standards other than those which are truly professional			− 0.219	− *0.474*	
	28	Violators of professional standards face fairly severe penalties		0.464			
	33	We really have no way of judging each other’s competence				− *0.521*	
	38	The professional organization is really powerless in terms of enforcing rules				− *0.439*	0.336
	43	There is not much opportunity to judge how another person does his work			− 0.468	− *0.329*	
	48	My colleagues pretty well know how well we all do in our work		0.445			
Factor 5: Belief in public service	2	Other professions are actually more vital to society than mine				0.627	*0.297*
	7	I think that my profession, more than any other, is essential for society		0.322			*0.284*
	12	The importance of my profession is sometimes over stressed		0.637			
	17	Some other occupations are actually more important to society than is mine				0.512	*0.363*
	22	Not enough people realize the importance of this profession for society			− 0.590		
	27	More occupations should strive to make a real contribution to society the way my own does					*0.313*
	32	Any weakening of the profession would be harmful for society					*0.707*
	37	The benefits this profession gives to individuals and society are underestimated					*0.471*
	42	It is impossible to say that any occupation is more important than any other	–	–	–	–	–
	47	If ever an occupation is indispensable, it is this one	0.351				

Loadings below 0.30 are suppressed, but items loading on factors from the original factors of Halls professionalism scale were retained even though they were lower than 0.30

Items loading on the original factors of Halls professionalism scale were italics

Six of the ten items of the factor, named ‘Sense of calling to the field’ loaded correctly, whereas 2 items loaded under ‘Belief in public service’. One item double-loaded: higher under ‘Using professional organisation as major referent’ and lower under ‘Belief in self-regulation’. Lastly, one item loaded under ‘Autonomy’.

Six of the ten items loaded correctly on factor 2, ‘Autonomy’; two items loaded higher under ‘Sense of calling to the field’; one item double-loaded higher under ‘Sense of calling to the field’ than ‘Belief in self-regulation’ and another under ‘Belief in self-regulation’.

Factor 3, ‘Using professional organisation as a major referent’, proved to be problematic because only 3 of the ten items loaded under this factor. One item loaded under ‘Sense of calling to the field’, one item double-loaded higher under ‘Sense of calling to the field’ than ‘Belief in public service’. Two items did not load above 0.30 on any factor. Another item double-loaded, higher under ‘Sense of calling to the field’ than ‘Autonomy’. There were also double-loadings under ‘Sense of calling to the field’ with the highest loading under ‘Belief in public service’. Another item loaded under ‘Belief in self-regulation’.

Seven of the ten items loaded correctly under, ‘Belief in self-regulation’, with one item loading under ‘Sense of calling to the field’ and two items under ‘Autonomy’.

Six of the ten items loaded correctly under, ‘Belief in public service’. One item loaded under ‘Autonomy’, one under ‘Using the professional organisation as major referent’, and one under ‘Sense of calling to the field’. One item had no loading above 0.30 on any factor. It was decided to perform a confirmatory factor analysis on these factors to determine the fit in a South African context.

##### Confirmatory factor analysis

**Fig. 1:**
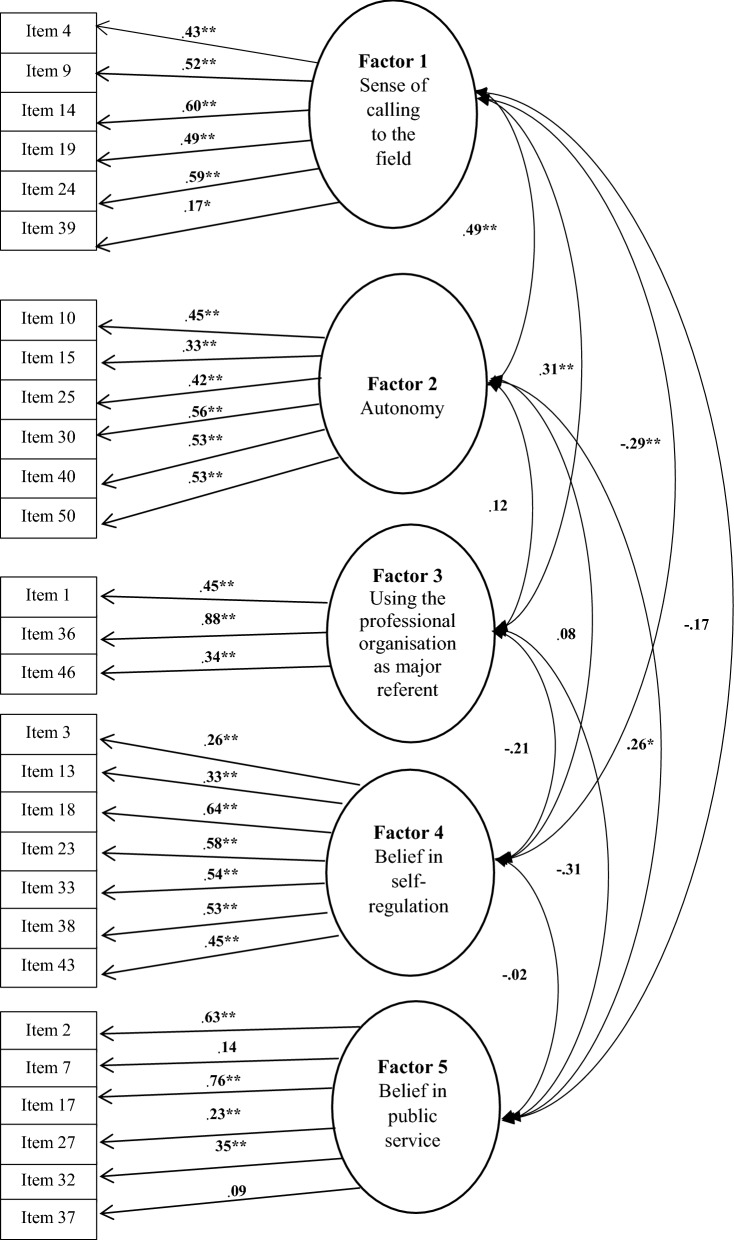
Confirmatory factor structure according to South African data. *Indicates statistical significance on a 10% level. **Indicates statistical significance on a 5% level

Figure 1 indicates items included in the South African model for confirmatory factor analysis.

All items loaded statistically significantly on a 5% level on the latent variables, and item 39 on a 10% level; while items 7 and 37 did not load statistically significantly. Factor 1, ‘Sense of calling to the field’, negatively correlated with both factor 4, ‘Belief in self-regulation’, and factor 5, ‘Belief in public service’, implying that the higher a person’s ‘Sense of calling to the field’, the lower his/her ‘Belief in self-regulation’ and ‘Belief in public service’. Factor 3, ‘Using the professional organisation as major referent’ correlated negatively with both factor 4, ‘Belief in self-regulation’, and factor 5, ‘Belief in public service’, indicating that the higher a person’s belief in ‘Using the professional organisation as major referent’, the lower his/her ‘Belief in self-regulation’ and ‘Belief in public service’.

The Chi square test is viewed as an overly strict indicator of model fit, given its power to detect trivial deviations from the proposed model [9]. The Chi square test statistic could be divided by degrees of freedom [10]. The factor model yielded a Minimum Sample Discrepancy divided by Degrees of Freedom (CMIN/DF) value of 1.80. Interpretation of the size of this value depends to some extent on the viewpoint of the investigator, but in practice a value of 2 indicates a good model fit [10]. It is, however, considered good practice to report multiple fit indices, typically from three broad classes [9]. The values above 0.9 indicate a good overall fit for a Comparative Fit Index (CFI) [10]. An unacceptable CFI of 0.59 was found for the model, while a Root Mean Square Error of Approximation (RMSEA) value of 0.07 with a 90% confidence interval of [0.062; 0.077] was obtained, which is considered acceptable. The models with RMSEA values of 0.10 and greater are unacceptable [11]. Although, two of the three fit indices indicate acceptable fit, it was decided to conduct a modified confirmatory factor analysis where items loading below 0.3 on their respective factors (items 3, 7, 27, 37 and 39) were removed.

After exclusion of these items, and the fit indices changed to a CMIN/DF value of 1.90, a CFI of 0.65, and the RMSEA value of 0.074 with a 90% confidence interval of [0.063; 0.084]. This also indicated that two of the three fit indices indicate acceptable fit, indicating construct validity in a South African context. However, rephrasing of currently excluded items and/or development of additional items for two factors namely factor 3, ‘Using the professional organisation as major referent’ and factor 5, ‘Belief in public service’, which both only included 3 items should be explored in future studies.

#### Reliability

The overall Cronbach’s alpha coefficient for the 23-item scale with South African data was 0.53. The acceptable Cronbach’s alpha for cognitive tests is 0.70, but when dealing with psychological constructs such as professionalism, values below 0.70 could realistically be predictable due to the diversity of the constructs being measured [6]. The Cronbach’s alpha for the sub-factors were acceptable, ranging between 0.52 and 0.67. The mean inter-item correlation was also determined, as this is advantageous when there are less than 10 items per factor [7]. The mean inter-item correlation should range between 0.15 and 0.55 [8]. The inter-item correlation ranged between 0.23 and 0.30, confirming reliability in the South African context. The means of ‘Belief in public service’ scored the lowest (2.16) while ‘Using the professional organisation as major referent’ and ‘Belief in self-regulation’ scored the highest (2.86) in the South African context (see Additional file 1: Table S1).

### Discussion

**Fig. 2:**
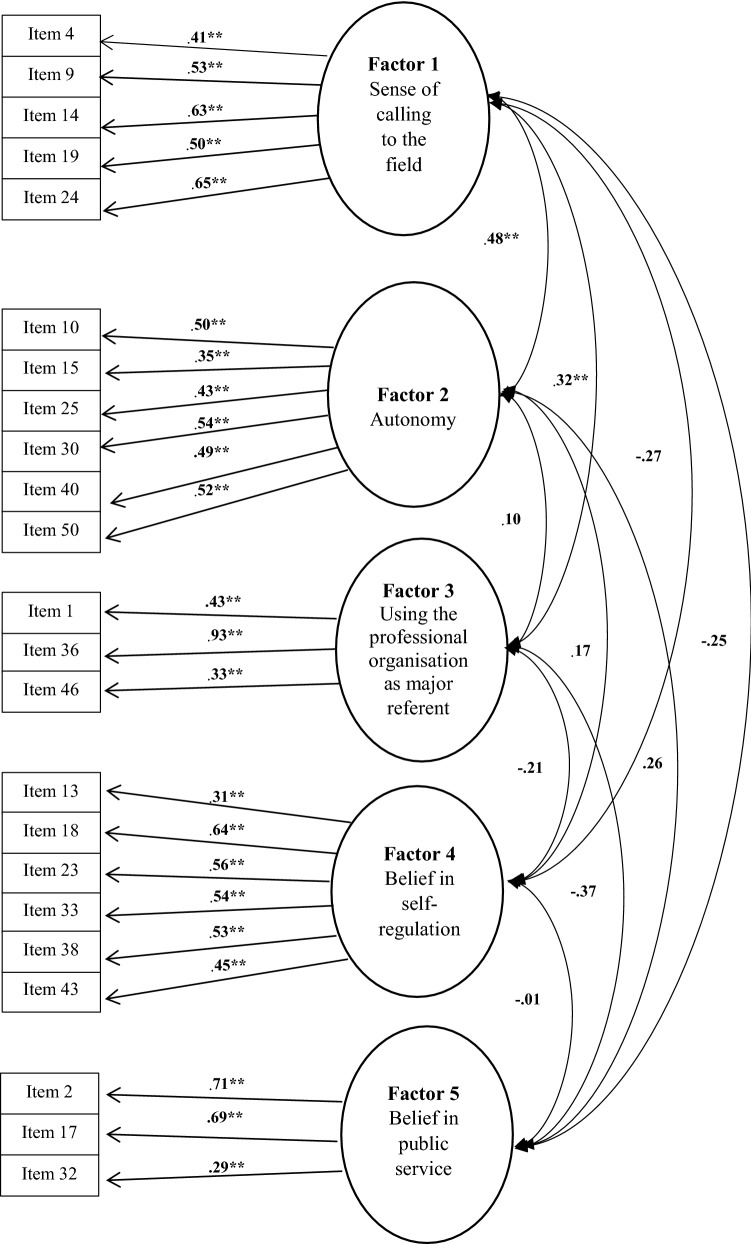
Modified factor structure according to South African data. **Indicates statistical significance on a 5% level

From the first preliminary South African study, it can be concluded that 23 items of HPS were sufficient for measuring the five attitudinal components of nurses’ professionalism. The confirmatory analysis (Fig. 1) indicated that 5 items (numbers 3, 7, 27, 37, 39) had loadings lower than 0.3, therefore a modified confirmatory factor analysis (Fig. 2) were done excluding these items. Satisfactory reliability of the HPS sub-scales were obtained, which included Cronbach’s alpha and mean inter-item correlations.

Five items (numbers 4, 9, 14, 19, 24) loaded under ‘Sense of calling to the field’; six (numbers 10, 15, 25, 30, 40, 50) under ‘Autonomy’; three (numbers 1, 36, 46) under ‘Using professional organisation as major referent’; six (numbers 13, 18, 23, 33, 38, 43) under ‘Belief in self-regulation’; and three (numbers 2, 17, 32) under ‘Belief in public service’. Two factors, namely factor 3, ‘Using professional organisation as major referent’ (numbers 1, 36, 46) and factor 5, ‘Belief in self-regulation’ (numbers 2, 17, 32), seemed to be the most problematic, as it only consisted of 3 items each with relatively lower internal consistency. South Africa is culturally diverse with 11 official languages. This led to the assumption that a number of the items in HPS could have been interpreted differently due to cultural misunderstandings. As a result, special attention should be given to developing items for these two factors in future studies with more participants.

## Limitations

- Data was collected from a specific group; therefore results could only be used as guide for the greater South African professional nurse population.
- A larger study should be conducted with more participants to test the construct validity and internal consistency of HPS.
- Usage of SMSs as data collection method is not conducive due to a low response rates.

## Additional file

**Additional file 1: Table S1.** Descriptive statistics and reliability of the final factors.

## Data Availability

The author can confirm that all relevant data are included in the article and/or its supplementary information files. The data is presented as supplementary files.

## Abbreviations

CFI: Comparative Fit Index
CIM/DF: Minimum Sample Discrepancy divided by Degrees of Freedom
HPS: Hall’s Professionalism Scale
RMSEA: Root Mean Square Error of Approximation
SMS: short message system
