# Supplementary material for: Construct validity and internal consistency of Hall’s Professionalism Scale: tested on South African nurses
Source: BMC Res Notes. 2019 Aug 6;12:486. doi: 10.1186/s13104-019-4515-6 (PMC6685167; doi:10.1186/s13104-019-4515-6)
Supplement: Supplementary file 1 — Additional file 1: Table S1. Descriptive statistics and reliability of the final factors. [file 13104_2019_4515_MOESM1_ESM.docx]

| **Table S1 Descriptive statistics and reliability of the final factors** | | | | |
| --- | --- | --- | --- | --- |
| **Factors** | **Cronbach alpha** | **Mean inter-item correlation** | **Mean** | **Standard Deviation** |
| **Factor 1:** Sense of calling to the field | 0.67 | 0.30 | 2.46 | 0.79 |
| **Factor 2:** Autonomy | 0.66 | 0.24 | 2.71 | 0.76 |
| **Factor 3:** Using the professional organisation as a major referent | 0.52 | 0.27 | 2.86 | 1.03 |
| **Factor 4:** Belief in self-regulation | 0.64 | 0.23 | 2.86 | 0.80 |
| **Factor 5:** Belief in public service | 0.58 | 0.30 | 2.16 | 0.95 |
